# Integrity at *Cancer Medicine*: the research we publish, how we evaluate it, and what we ask of our authors

**DOI:** 10.1002/cam4.8

**Published:** 2012-07-17

**Authors:** Chris Graf, David L Vaux

**Affiliations:** 1 Wiley-BlackwellRichmond, Victoria, Australia; 2 The Walter and Eliza Hall InstituteParkville, Victoria, Australia

*Cancer Medicine* aims to publish novel, scientifically sound, research that advances our understanding of the occurrence, causes, diagnosis, and treatment of cancer. As an open access publication, articles appearing in *Cancer Medicine* will be available to everyone, free of charge. This is made possible by authors, institutions, and research funders paying an article publication charge [1]. To provide equal access to authors regardless of nationality or affiliation, articles submitted directly to *Cancer Medicine* will be assessed by double-blind peer review.

Scientific progress depends on the integrity of research publications. The editors of *Cancer Medicine* aim to establish and maintain high standards of data integrity and publication ethics in all the papers we publish. To do this, we have set detailed guidelines for authors and reviewers [1], and we will monitor compliance with those guidelines.

## Double-blind peer review and our attempts to reduce bias and provide equal opportunity to publish

All research articles published in *Cancer Medicine* will undergo full peer review.

- Research articles submitted directly to *Cancer Medicine* will initially be evaluated by the Editor-in-Chief; articles found to fit with the scope of the Journal will be reviewed by at least two suitably qualified experts.
- Manuscripts submitted directly to *Cancer Medicine* will be subject to “double-blind” peer review: the names of the authors, their addresses, and affiliations will not be revealed to the reviewers.
- Most submissions transferred to *Cancer Medicine* from partnering journals will be assessed using the original peer-review reports from those journals; however, the Editor-in-Chief will critically review these peer-review reports and may choose to send manuscripts out for additional double-blind review.
- All publication decisions are made by the Editor-in-Chief on the basis of the reviews provided.
- Members of the editorial board lend insight, advice, and guidance to the Editor-in-Chief generally and assist in decision making on specific submissions. When the Editor-in-Chief requests advice from editorial board members about a specific manuscript, the names of the authors, their affiliations, and nationalities will not be revealed to the editorial board members.
- The managing editor and editorial assistant provide administrative support to ensure that *Cancer Medicine* maintains the integrity of peer review and delivers rapid and efficient publication to authors and reviewers.

## Errors

It is important to correct errors, whether they are inadvertent or deliberate, because they can lead to inappropriate allocation of credit, waste researchers’ time and resources, and may even put patients at risk.

If readers raise concerns with us, we will take action promptly, in accordance with the guidelines from the Committee on Publication Ethics [2]. Although accidental errors can usually be handled easily by publishing a correction, cases of fabrication, falsification, or plagiarism are always more challenging for editors and publishers. This is because, at least in the first instance, editors only communicate with the corresponding author, and the primary data resides at the host institution. Therefore, resolving such cases often depends on a cooperative effort by both the journal and the research institution. Where authors request a paper to be retracted, or after a suitable investigation an institution requests a paper to be retracted, *Cancer Medicine* will publish the retraction, and include a full explanation for why it is doing so.

## Images

Instructions for preparation of figures are set out in the Author Guidelines [1]. In addition, prior to final acceptance, authors must agree to submit the source data (e.g., uncropped high-resolution scans of gels and blots) if requested. Failure to provide source data on request will result in rejection of the manuscript.

## Checklists for submission

During online submission, authors must indicate that:

- all authors have seen the submitted manuscript, and the nature of their contributions are indicated;
- all those who have contributed to the publication are listed either as authors or in the acknowledgments;
- all funding sources are acknowledged;
- potential conflicts of interest are declared;
- primary source data will be provided on request for papers that are accepted for publication;
- experiments were performed in accordance with the relevant human and animal ethics regulations, and the relevant ethics committees are named;
- clinical trials are registered with the appropriate body.

## Notifications

All authors will be notified by *Cancer Medicine* on submission of a new manuscript.

## Experimental materials and data sharing

Authors must make available to other researchers, or provide alternative sources, for all novel reagents described in the experiments. This includes viruses, cells, nucleic acids, and antibodies. Experimental organisms (e.g., genetically modified mice) must also be made available, but if this is impracticable, the means to reproduce them (e.g., ES lines, transgenic constructs) must be provided. Human patient samples and data should be made available in accordance with the relevant ethical standards. Costs incurred in producing and transferring the materials should be borne by those who make the request, and provision would be subject to Materials Transfer Agreements at the discretion of the provider. *Cancer Medicine* will only publish manuscripts if the authors agree to make all data including novel nucleotide sequences, structural data, or data from large-scale gene expression experiments freely available, where possible in public databases [3].

## Screening of text and figures

The text of all submitted manuscripts will be submitted to CrossCheck to detect matching or overlapping text, in an effort to prevent publication of materials that may have been plagiarized. Appropriate action will be taken on manuscripts where CrossCheck identifies a high percentage of similarity or overlap with previously published materials or other materials identified on the Internet. All figures in accepted papers will be screened for compliance with the author guidelines prior to publication.

## After publication

If authors become aware of an error after publication, or if readers have concerns that errors may exist, they are encouraged to write to the editors of *Cancer Medicine*. If the authors agree the correction, we will endeavor to publish it quickly. If there is no agreement over the nature of an error, the authors are in dispute, or cannot provide a satisfactory explanation, the editors will handle the matter in accordance with the COPE flowcharts, which will most likely involve alerting representatives from the host institution, and possibly the relevant national office or ombudsman for research integrity.

## Feedback welcomed

The research integrity policies of *Cancer Medicine* have been based in part on those of other journals, and we have tried to build on the experience of COPE and blogs such as Retraction Watch [4]. We encourage suggestions for further improvement from our readers to cancermed@wiley.com.

## Disclosures

C. G. works for John Wiley & Sons, and benefits from the success of the company's publishing program. C. G. publishes clinical and research journals including a number for Societies and Royal Colleges in Australia and New Zealand, the *International Journal of Clinical Practice*, the global Wiley Open Access journals in health sciences, is Treasurer of Committee on Publication Ethics (a UK-registered charity), and leads the Wiley-Blackwell publication ethics program. D. L. V. sits on the editorial board of *Cancer Medicine*.
